# Safety and Optimization of Metabolic Labeling of Endothelial Progenitor Cells for Tracking

**DOI:** 10.1038/s41598-018-31594-0

**Published:** 2018-09-04

**Authors:** Sang-Soo Han, Hye-Eun Shim, Soon-Jung Park, Byoung-Chul Kim, Dong-Eun Lee, Hyung-Min Chung, Sung-Hwan Moon, Sun-Woong Kang

**Affiliations:** 1 Predictive Model Research Center, Korea Institute of Toxicology, Daejeon, Korea; 2 Department of Stem Cell Biology, School of Medicine, Konkuk University, Seoul, Korea; 3 The Genomics Institute, Ulsan National Institute of Science and Technology, Ulsan, Korea; 4 Advanced Radiation Technology Institute, Korea Atomic Energy Research Institute, Jeonbuk, Korea; 5 Department of Human and Environmental Toxicology, University of Science and Technology, Daejeon, Korea

## Abstract

Metabolic labeling is one of the most powerful methods to label the live cell for *in vitro* and *in vivo* tracking. However, the cellular mechanisms by modified glycosylation due to metabolic agents are not fully understood. Therefore, metabolic labeling has not yet been widely used in EPC tracking and labeling. In this study, cell functional properties such as proliferation, migration and permeability and gene expression patterns of metabolic labeling agent-treated hUCB-EPCs were analyzed to demonstrate cellular effects of metabolic labeling agents. As the results, 10 μM Ac4ManNAz treatment had no effects on cellular function or gene regulations, however, higher concentration of Ac4ManNAz (>20 μM) led to the inhibition of functional properties (proliferation rate, viability and rate of endocytosis) and down-regulation of genes related to cell adhesion, PI3K/AKT, FGF and EGFR signaling pathways. Interestingly, the new blood vessel formation and angiogenic potential of hUCB-EPCs were not affected by Ac4ManNAz concentration. Based on our results, we suggest 10 μM as the optimal concentration of Ac4ManNAz for *in vivo* hUCB-EPC labeling and tracking. Additionally, we expect that our approach can be used for understanding the efficacy and safety of stem cell-based therapy *in vivo*.

## Introduction

Human umbilical cord blood-derived endothelial progenitor cells (hUCB-EPCs) have a great potential therapeutic impact in clinical trials of acute nervous system disorders, myocardial infarction and stroke^1–3^. hUCB-EPCs have been also used to investigate the repair of injured vessels and neovascularization or regeneration of ischemic tissues and have been used for therapeutic re-endothelialization of vein graft because of their ability to induce neovascularization under ischemic conditions^4–6^. Moreover, hUCB-EPC can be obtained without extensive surgical procedure^7^ and are immediately available, there are no risks to the donor and there is a low risk of transmitting infectious diseases^8^. For these reasons, currently, hUCB-EPCs are the preferred tool for animal- and patient-based studies than other forms of pluripotent hematopoietic stem cells and mesenchymal stem cells for transplantation^9–11^. However, many unknown factors, including their regenerative property, the fate of transplanted hUCB-EPCs, *in vivo* migration to the site of injury and *in situ* differentiation have yet to be exploited in more efficient ways to treat various diseases^12–14^.

For understanding biological mechanisms and the therapeutic effects of inoculated cells *in vivo*, cell labeling and tracking are very useful processes^15^. Although direct labeling with specific dyes or indirect labeling with genetic cell modifications and reporter genes^16^ have been used to observe unknown factors of transplanted hUCB-EPCs. These labeling methods have several safety and technical issues, such as photobleaching, quenching, sensitivity to pH changes and multiple labeling steps^17^. Recently, various techniques, including fluorescence, bioluminescence, positron emission tomography (PET), single photon emission computed tomography (SPECT) and magnetic resonance imaging (MRI), have been developed and used for hUCB-EPC labeling and tracking^18–21^. Many studies have used MRI methods with iron oxide- and ^19^F-based probes^22–25^. Wang *et al*. and Willenbrock *et al*. reported that magnetically labeled hUCB-EPCs in a mouse model were detectable for more than 7 days post-transplantation and have many advantages such as a simple and low-cost labeling, an effective imaging window and good signal intensity^26,27^. However, MRI labeling is limited by the persistence of the *in vivo* signal even after death of the transplanted cells, such that the MRI signal does not correlate with the viability of transplanted cells^28^.

Metabolic labeling is the preferred labeling technique for tracking live cells because it has many advantages such as low background, correlation of cell survival and simple steps for cell labeling^29,30^. In addition, the reaction produces very few toxic and non-toxic byproducts and therefore, metabolic labeling has advantages that apply to *in vivo* studies. Till now, the metabolic labeling technique in stem cells has been mostly used for detecting glycoprotein markers of mesenchymal stem cell differentiation^31^, for identifying or isolating live colon cancer stem cells^32^ and for quantifying protein abundance in embryonic stem cells (ESCs)^33^. However, the cellular mechanisms by which modified glycosylation due to metabolic agents are not completely understood^34^. Recently, Lee. *et al*. reported that the metabolic labeling technique can stably label stem cells, but this study only focused on efficient labeling of stem cells^35^.

In this study, to validate the safety and optimize the metabolic labeling method, we analyze cell functional properties, such as proliferation, migration and permeability and gene expression patterns of metabolic labeling agents-treated hUCB-EPCs. First, we screened the azido sugars for more efficient labeling of hUCB-EPCs. And then, we observed changes in basic cellular events including cell growth, migration, permeability and mitochondrial function. We analyzed transcriptomic changes by RNA-seq, functional characterization of hUCB-EPCs by observation of tube formation and marker gene expression using flow cytometric analysis.

## Results

### Screening of metabolic labeling agent for efficient labeling of hUCB-EPCs

Metabolic labeling method was described as the introduction of subtle modifications into monosaccharide precursors, such as in the case of introducing an azido group from azide-functionalized monosaccharides (tetra-acetylated N-azidoacetylmannosamine (Ac4ManNAz), tetra-acetylated N-azidoacetylgalactosamine (Ac4GalNAz), or tetra-acetylated N-azidoacetylglucosamine (Ac4GlcNAz)) via post-translational modification (PTM)^36^. Once the azido group was introduced into cellular glycans, the glycosylation position and type of three azide-functionalized monosaccharides differed based on each sugar type^37^. In detail, the Ac4ManNAz was metabolically converted to azido sialic acid derivative, which is used for N-linked glycosylation of cell surface proteins^38^, whereas Ac4GlcNAz and Ac4GalNAz were predominantly used for O-linked glycosylation as a substitute for O-GlcNAc and O-GalNAc, which are attached to serine and threonine side chain of numerous intracellular proteins^39^. When using metabolic labeling reagents, the choice of reagents was found to be very important for efficiently labeling the target cells. Thus, we first screened the metabolic labeling reagents for efficient labeling hUCB-EPCs.

**Figure 1:**
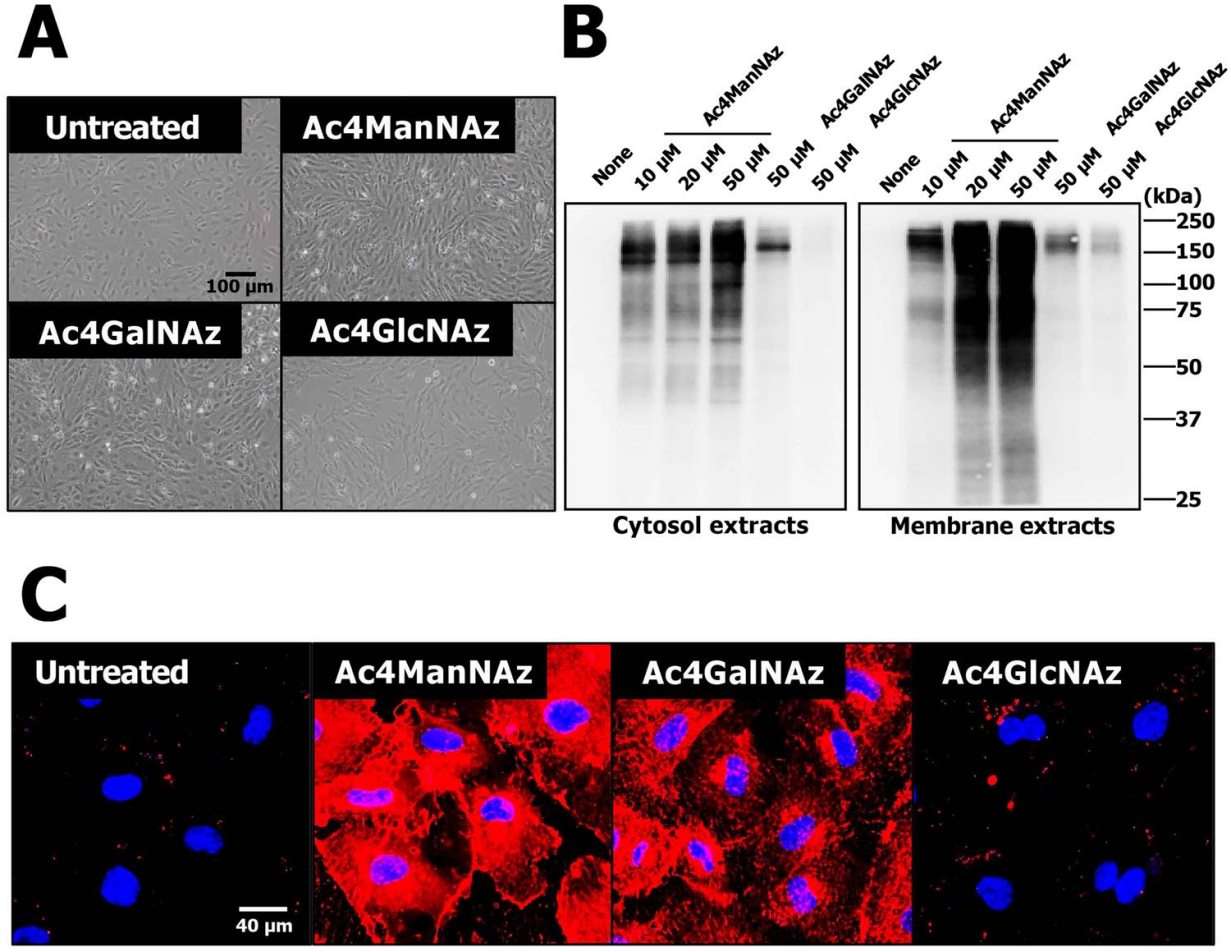
Screening metabolic labeling agents for hUCB-EPC labeling and tracking. (**A**) Morphological properties of hUCB-EPCs treated with three metabolic labeling agents (Ac4ManNAz, Ac4GlcNAz and Ac4GalNAz) were analyzed using microscopic observation. (**B**) Western blot analysis of metabolic labeling agent-treated hUCB-EPCs showing total proteins and generated azide groups. In the figure indicated full-length expression and all gels were run under the same experimental conditions while images of western blots displayed. (**C**) Visualization of metabolic labeling agent-treated hUCB-EPCs using DBCO-cy5 (n = 3).

Isolated hUCB-EPCs were treated with 10, 20, or 50 μM Ac4ManNAz, Ac4GlcNAz, or Ac4GalNAz, which can easily access the modified surface and intracellular proteins with the azido group via PTM. hUCB-EPCs treated with three azido sugars did not show changes or differences in cell morphology (Fig. 1A). To analyze the incorporation of azido groups, Western blot analysis was performed as described in materials and methods. Interestingly, Ac4ManNAz treatment resulted in a higher generation efficiency of the azido group than Ac4GalNAz and Ac4GlcNAz treatment (Fig. 1B). Azido groups were incorporated into cytosolic and membrane proteins on hUCB-EPCs treated with Ac4ManNAz and the rate of incorporation of labeled proteins gradually increased relative to Ac4ManNAz concentration. However, hUCB-EPCs treated with 50 μM Ac4GalNAz or Ac4GlcNAz generated only the modified proteins and Ac4GlcNAz treatment labeled membrane proteins. In addition, the generation of azido groups was determined by biorthogonal copper-free click chemistry with dye-labeled dibenzyl cyclooctyne (DBCO-Cy5) (Fig. 1C). Ac4ManNAz treatment resulted in higher labeling efficiency than Ac4GalNAz and Ac4GlcNAz, which was similar to results from the Western blot analysis. Thus, we finally selected Ac4ManNAz for metabolic labeling of hUCB-EPCs.

### Bio-physiological effects of Ac4ManNAz-treated hUCB-EPCs

When hUCB-EPCs were treated with Ac4ManNAz, an abiotic azido group was introduced to native proteins by PTM. Additionally, Ac4ManNAz, which is an analog of ManNAc, was used as a carbon source^40^. Generally, the carbon source concentration can impair many cellular functions such as cell proliferation, viability and permeability^41,42^. Additionally, glycosylation of native proteins regulates their functions and kinetics and changes in glycosylation can alter cellular functions such as host cell surface interactions and modulation of cell signaling and gene expression^43^. Thus, Ac4ManNAz labeling of hUCB-EPCs still introduces the possibility of altering cellular physiology.

**Figure 2:**
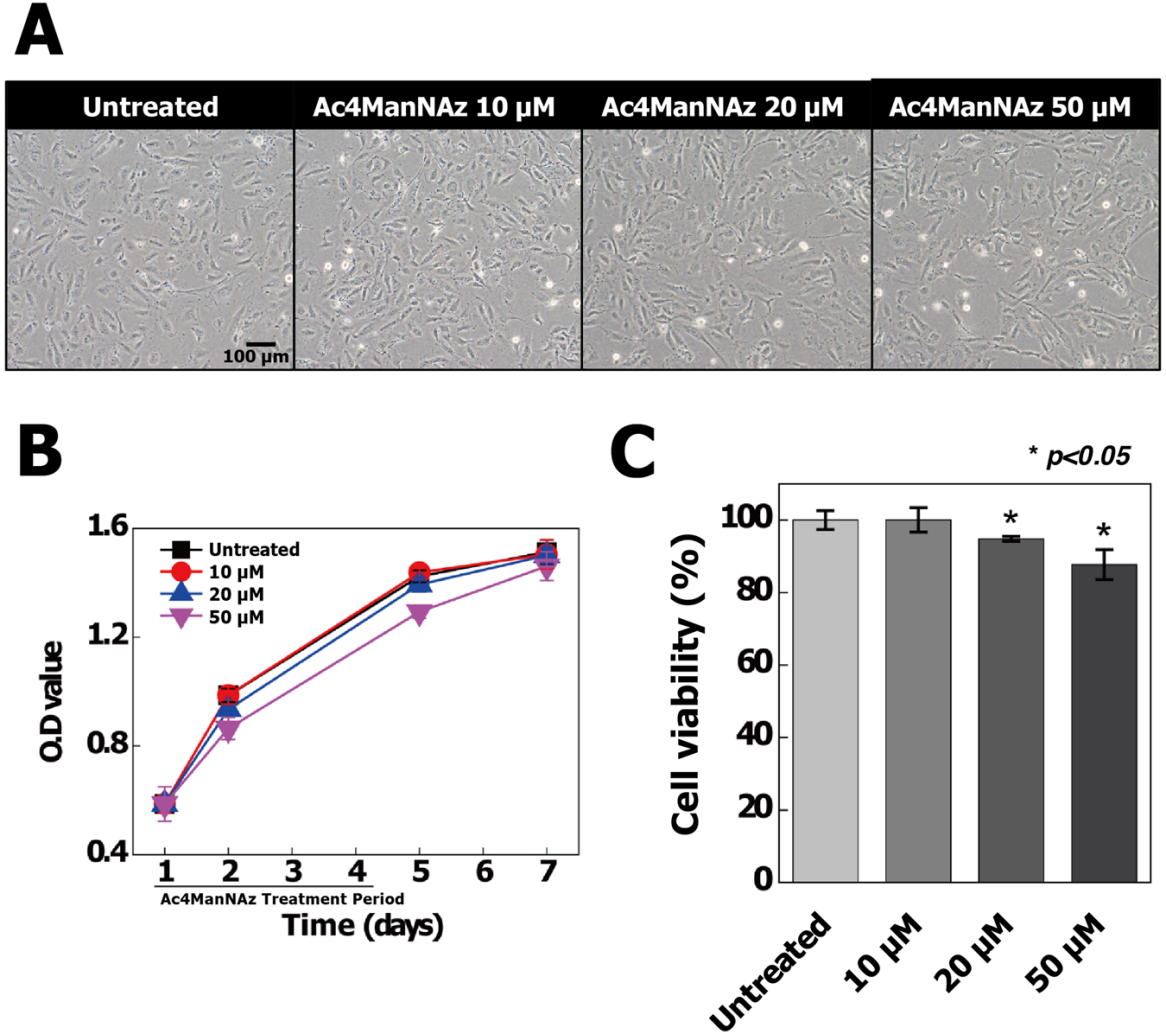
Analysis of morphological properties, proliferation ability and viability of Ac4ManNAz-treated hUCB-EPCs. All hUCB-EPCs were incubated with various concentrations of Ac4ManNAz (0 to 50 µM). (**A**) Ac4ManNAz concentration-dependent morphological changes were analyzed by microscopic observation. Cell growth rate (**B**) and viability (**C**) were analyzed by CCK-8 and manual microscopic counting.

**Figure 3:**
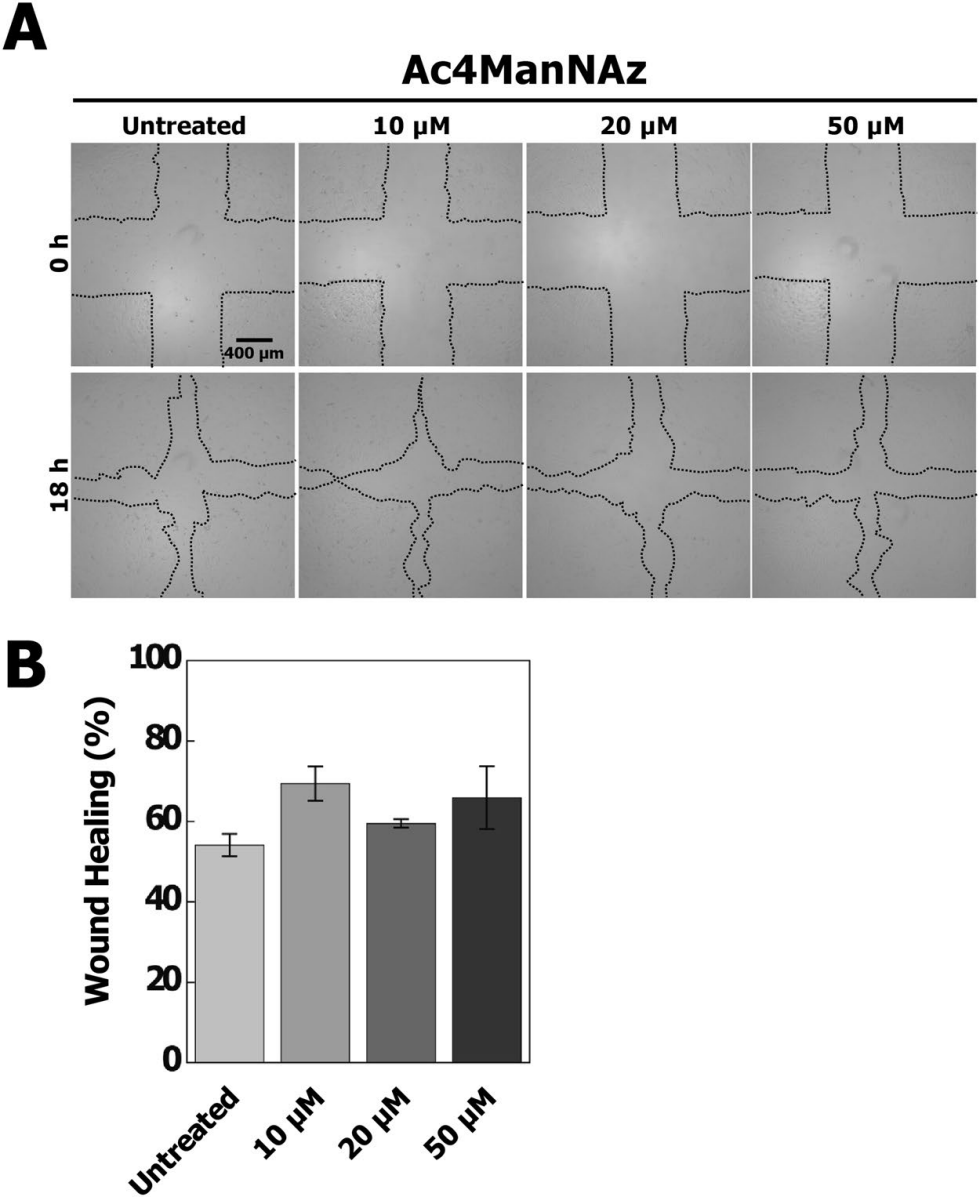
Wound healing assay in Ac4ManNAz-treated hUCB-EPCs. Wound healing assay was performed to assess the effect of Ac4ManNAz on the migration of hUCB-EPCs. The assay was repeated three times and representative images (**A**) and quantification (**B**) are shown.

**Figure 4:**
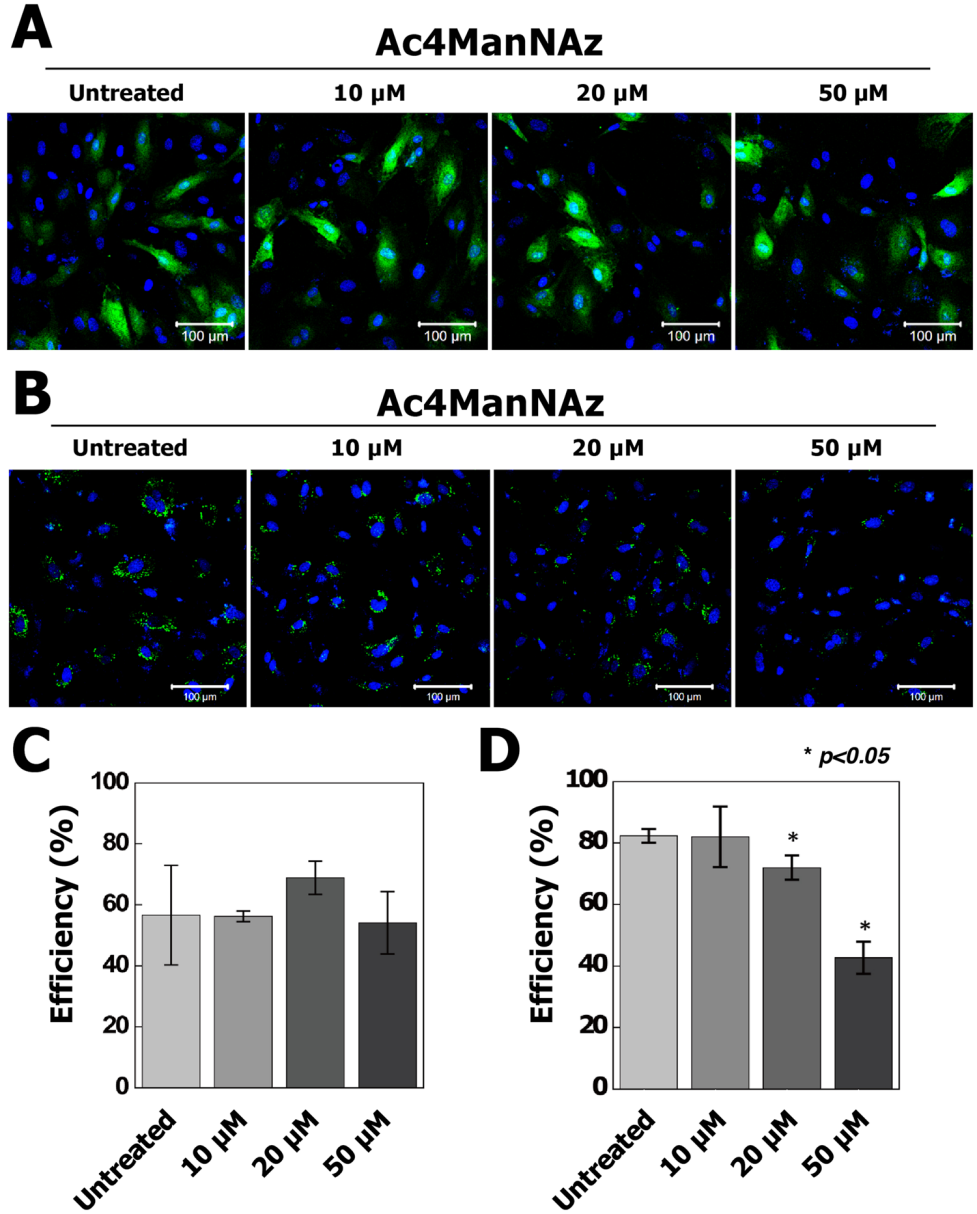
Analysis of cell permeability via transfection and Qdot 525 labeling in Ac4ManNAz-treated hUCB-EPCs. (**A**) Ac4ManNAz-treated hUCB-EPCs were transfected with pcDNA3-eGFP and GFP fluorescence and analyzed using fluorescence microscopy. (**B**) Confocal fluorescence images of live hUCB-EPC labeling with a Qdot 525 probe (green). Quantification of the transfection (**C**) and Qdot labeling (**D**) efficiency was conducted.

To validate the influence of Ac4ManNAz on hUCB-EPCs, we firstly performed cytotoxic tests using CCK-8 assay and cell morphology assessment. Morphological study suggests that treatment of Ac4ManNAz did not affect significantly on cell morphology (Fig. 2A). However, the growth rate gradually decreased in hUCB-EPCs treated with >20 µM Ac4ManNAz (Fig. 2B) and cell viability data showed that treatment of >20 µM Ac4ManNAz significantly decreased cell viability, approximately 6.2% and 12.3% (*p < 0.05) at the concentrations 20 µM and 50 µM of Ac4ManNAz as compared to control (Fig. 2C). We furthermore examined the effects of Ac4ManNAz using *in vitro* wound healing assay. The scratch wounds were almost the same size in each experimental group at 0 h; after 18 h, the difference in the reduction in wound size was not statistically significant in any Ac4ManNAz (10, 20 and 50 µM) treatment group (Fig. 3). In addition, we analyzed the permeability of hUCB-EPCs treated with Ac4ManNAz using liposome-mediated transfection of a pcDNA3-eGFP plasmid and Qdot 525 probe. The results of the permeability test using eGFP showed no significant differences between the treated and untreated conditions (Fig. 4A,C). However, results from the Qdot analysis revealed a gradual decrease in the rate of endocytosis of hUCB-EPCs treated with >20 µM Ac4ManNAz (Fig. 4B,D). These results suggested that the membrane fusion was not changed; however, the rate of endocytosis was reduced in cells treated with >20 µM Ac4ManNAz.

**Figure 5:**
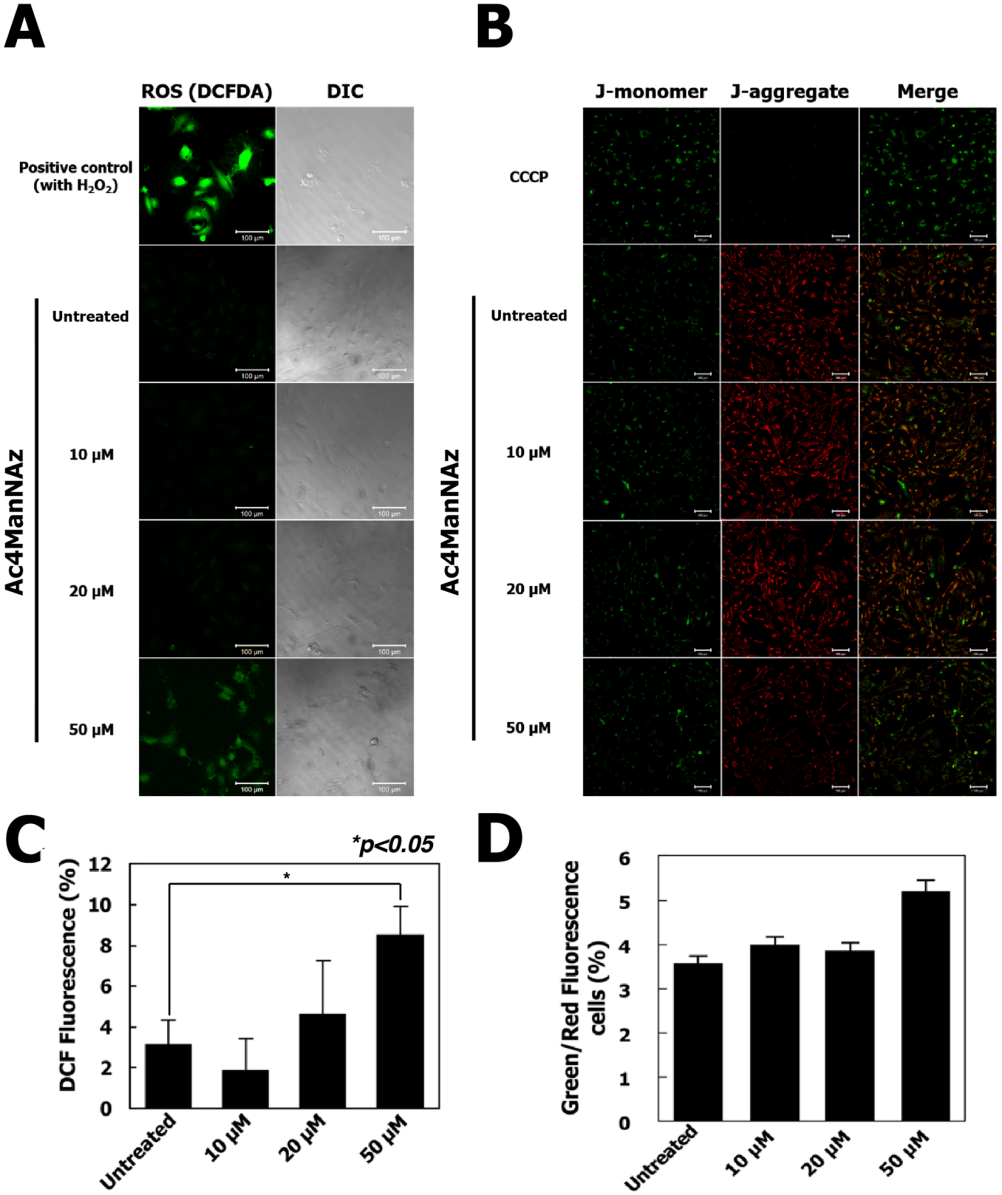
Analysis of reactive oxygen species (ROS) production and mitochondrial function in hUCB-EPCs treated with Ac4ManNAz. (**A**) hUCB-EPCs were treated with 0, 10, 20, or 50 μM Ac4ManNAz, and intracellular ROS levels were then measured using the fluorescent probe 2′, 7′-dichlorfluorescein-diacetate (DCFH-DA). (**B**) Changes in mitochondrial membrane potential (ΔΨm) were measured in hUCB-EPCs using the fluorescent probe JC-1. (**C**) Quantification of ROS generation are expressed as the percentage of fluorescence intensity relative to the control. (**D**) Value of mitochondrial membrane potential were expressed as % Green/Red fluorescence cells.

Additionally, we conducted the reactive oxygen species (ROS) generation assay and assessment of mitochondrial membrane potential (ΔΨm) to analyze the apoptosis induction by Ac4ManNAz treatments. The Fig. 5A suggests that hUCB-EPCs treated with 50 µM Ac4ManNAz significantly increased ROS intensity as compared to control. The quantitative measurement of ROS intensity was sustained increasingly to approximately 5.4% (*p < 0.05) at the concentrations 50 µM of Ac4ManNAz (Fig. 5C). The increased and decreased florescent intensity of red and green florescent caused by JC-1 indicates the change in mitochondrial membrane potential (ΔΨm). Result indicated that hUCB-EPCs treated with 50 µM of Ac4ManNAz reduced a red fluorescence (Fig. 5B). As shown from quantitative data, the Green/Red-fluorescence cells ratio were increased to 1.5% at 50 µM of Ac4ManNAz treatment (Fig. 5D). Although increased ROS generation and reduced of JC-1 red fluorescence indicated potent apoptotic activity of treatment of 50 µM Ac4ManNAz, these changes are not clear which of them are directly associated with hUCB-EPCs apoptosis. To prove this, we additionally analyzed the apoptotic effects of Ac4ManNAz by Annexin V staining (Fig. S1). As the results, apoptosis rates of hUCB-EPCs treated with higher concentration (more than 20 μM) of Ac4ManNAz were not changed compared with non-treated hUCB-EPCs. These results described that high concentration of Ac4ManNAz slightly modulated the generation of ROS and mitochondrial membrane potential, but these changes were not induced apoptosis.

### Transcriptomic change in Ac4ManNAz-treated hUCB-EPCs

**Figure 6:**
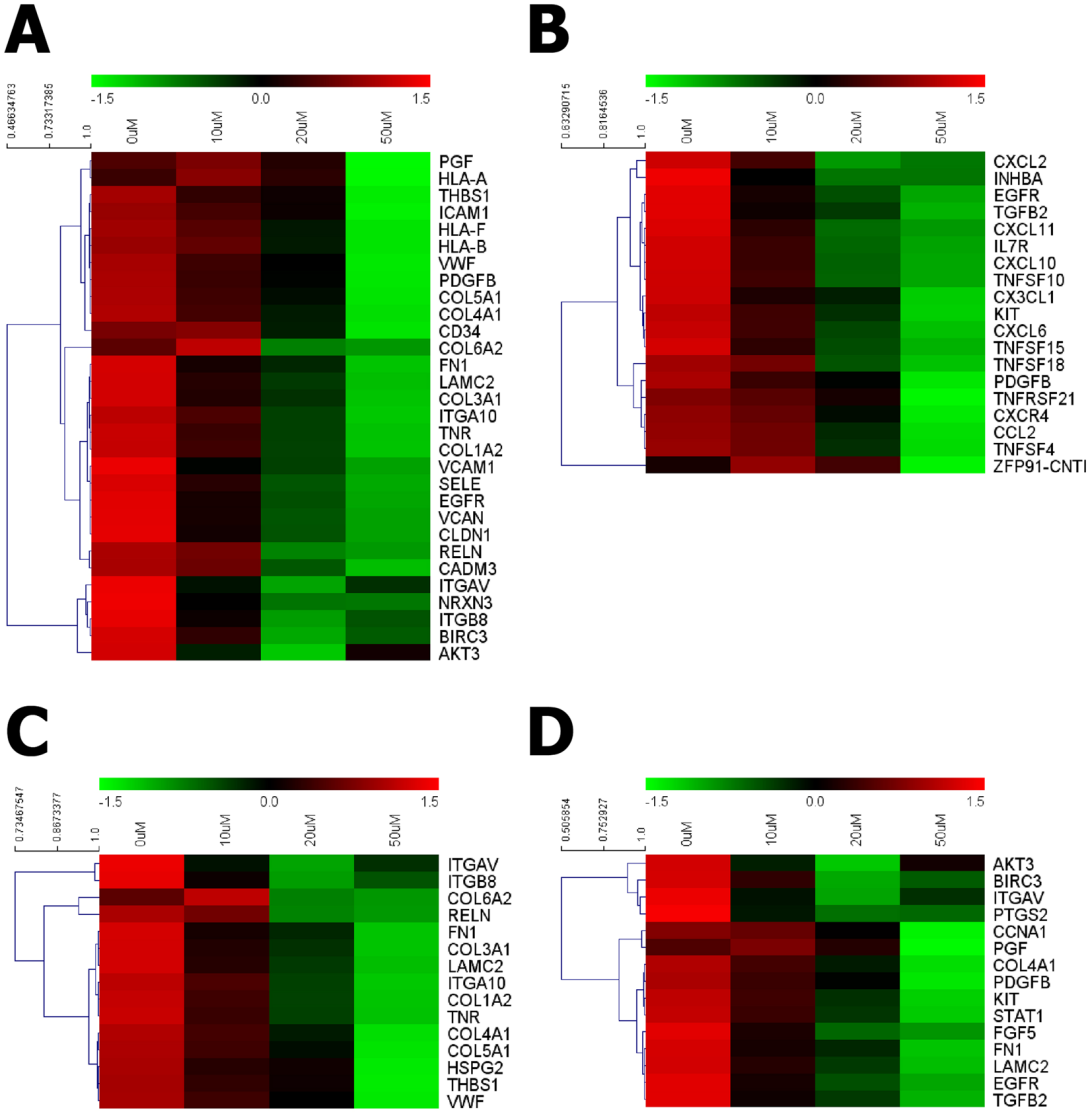
Transcriptomic analysis of effects of Ac4ManNAz treatment on hUCB-EPCs. Expression profiles of 0, 10, 20, and 50 μM Ac4ManNAz-treated hUCB-EPCs are shown. Heat map representation of mapped reads corresponds to protein-coding genes and the bio-functional pathway. (**A**) Cell adhesion, (**B**) cytokine-cytokine receptor interaction, (**C**) ECM-receptor interaction and (**D**) cancer-related pathways.

To additionally address the metabolic labeling effects, we explored the transcriptome patterns of hUCB-EPCs treated with various concentrations of Ac4ManNAz. mRNA from hUCB-EPCs treated with 10, 20 and 50 μM of Ac4ManNAz and untreated hUCB-EPCs was extracted and analyzed using the HiSeq2000 platform. Next, based on the expression levels of known genes, we identified 615 genes that were changed by 2-fold or more with a threshold of “*p* < 0.05 and FDR < 5”. And then, to identify cellular functional process-related genes, we carried out GO term analysis. Interestingly, in accordance with the increase in Ac4ManNAz concentration on hUCB-EPCs, 468 genes related to cell adhesion, cytokine-cytokine receptor interaction, extracellular matrix (ECM)-receptor interaction and cancer pathway were down-regulated (Fig. 6).

Of note, treatment with 20 and 50 µM Ac4ManNAz resulted in considerable down-regulation of many genes in the cell functional pathways of hUCB-EPCs. In cell adhesion, genes related to primary interactions with the cell matrix (LAMC3, ITGA5, ITGA10, ITGB8, TNR, EGFR and RELN) and integrin (CAMs) (ICAM1 and VCAM1), primary roles in cell-cell interactions (SELE), cell-cell interaction-mediated MHC (HLA-B, HLA-F and CD34) and collagen proteins (COL1A1, COL3A1, COL4A1, COL5A1 and COL6A2) were significantly down-regulated (Fig. 6A). In addition, genes related to cytokine-cytokine receptor interaction and ECM-receptor interaction were also down-regulated. In detail, gene expression levels of chemotactic cytokines (chemokines) (CCL2, CX3CL1, CXCL2, CXCL6, CXCL10, CXCL11 and CXCR4), tumor necrosis factors (TNFs) (TNFSF4, TNFSF10, TNFSF15, TNFSF18 and TNFRSF21), integrins (ITGA5, ITGA10, ITGB8) and ECM proteins (FN1, TNR and THBS1) were reduced by increasing the Ac4ManNAz concentration (Fig. 6B,C). Additionally, genes related to the regulation of cell cycle (CCNA1), cell growth, proliferation, apoptosis and the immune response (AKT3, STAT1, BIRC3, PTGS2, KIT, EGFR) were down-regulated (Fig. 6D). These results showed that Ac4ManNAz regulated a wide range of cell adhesion pathways and cell physiological pathways. In particular, these results also suggest that treatment >20 µM Ac4ManNAz induces the immune response and inhibits cell cycle progression, cell proliferation and cell adhesion. Interestingly, contrary to previous reports, Ac4ManNAz modulated cell biological functions.

### Analysis of hUCB-EPC functions after Ac4ManNAz treatment

In our results regarding the effects of metabolic labeling on hUCB-EPCs, higher concentrations of Ac4ManNAz negatively affected cell growth, proliferation, adhesion and rate of endocytosis and induction of ROS generation and mitochondrial depolarization. Of note, results from the gene expression analysis revealed the down-regulated expression of integrins, which are important for stem cell proliferation and self-renewal regulated via PI3K and focal adhesion kinase (FAK) signaling pathways^44^. Additionally, AKT3 gene, which is downstream of the PI3K and FAK signaling pathways^45^, was down-regulated. However, interestingly, there was no significant change in the gene expression of cadherin, which is one of the most important molecules in stem cell pluripotency and stemness^46,47^. Stem cells are known to have two important characteristics that distinguish them from other types of cells^48^. First, all stem cells are unspecialized. Second, under certain biochemical cues, stem cells can be induced to differentiate. Thus, as it was unclear whether Ac4ManNAz affects hUCB-EPC functions, we conducted the tubule formation assay and immunophenotyping of marker genes to analyze these important characteristics of EPCs.

**Figure 7:**
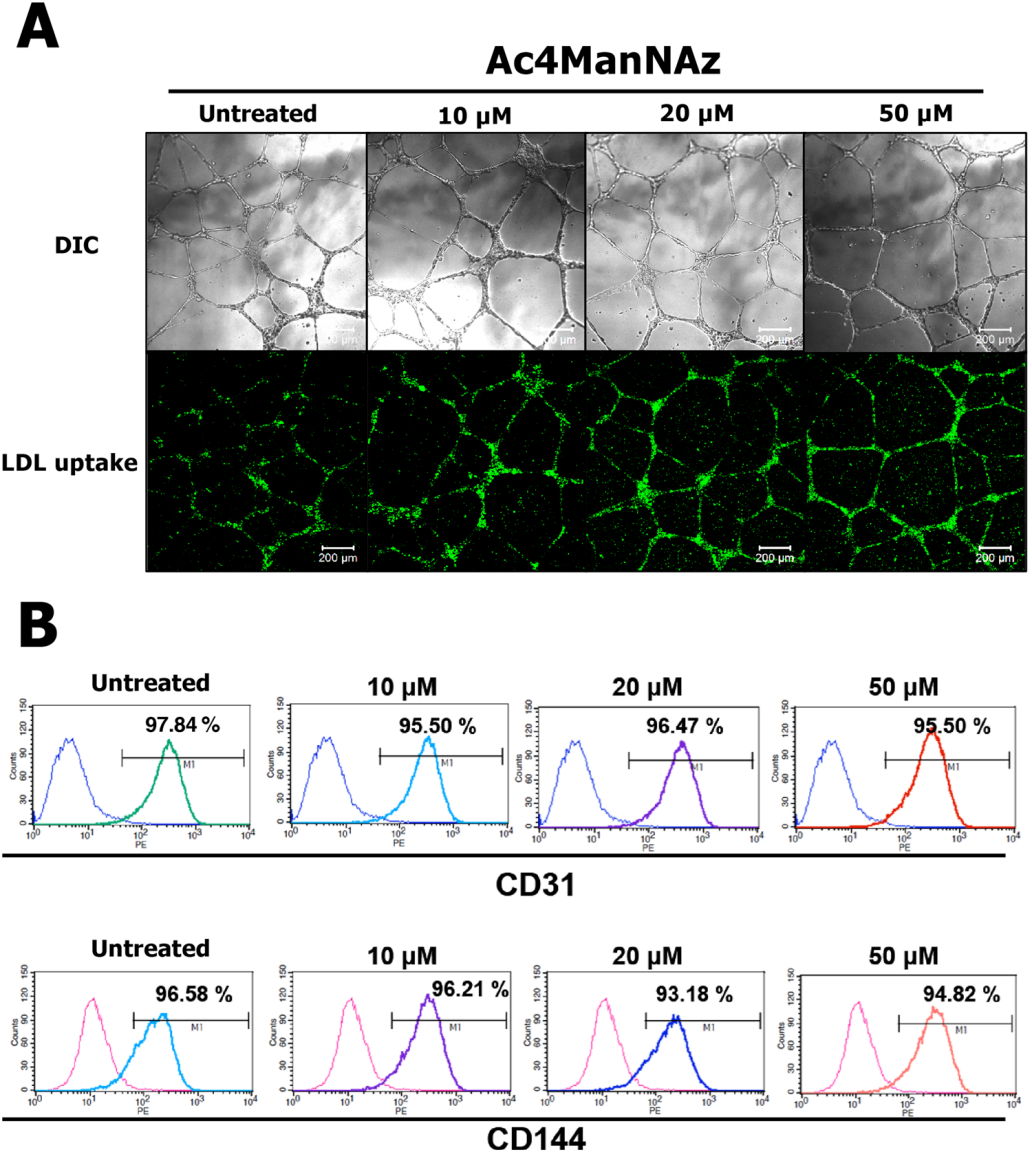
Analysis of tube formation and LDL uptake assay of Ac4ManNAz-treated hUCB-EPCs. (**A**) hUCB-EPCSs treated with 0, 10, 20, or 50 μM Ac4ManNAz were transferred to Matrigel-coated plates and cell rearrangement and tube structure formation were captured by a light microscope equipped with digital charge-coupled device camera after 48 h. Images of ac-LDL uptake were captured by fluorescence microscopy. (**B**) Representative FACS analysis demonstrated the function of hUCB-EPC by their markers CD31 and CD144.

To define the effects of Ac4ManNAz on hUCB-EPC functions, we carried out a tube formation assay on Matrigel to determine the angiogenic potential. We observed tube-like structures 4 days after treating hUCB-EPCs with 0, 10, 20 or 50 µM Ac4ManNAz. The upper of Fig. 7A shows typical tube formation in all samples. One of the hallmarks of hUCB-EPCs is their ability to internalize ac-LDL via the “scavenger cell pathway” of LDL metabolism^49^. At the endpoints of each protocol, we incubated our endothelial cultures with 1 μg/mL ac-LDL conjugated with a fluorescent dye (Fig. 7A lower). In addition, the expression of specific endothelial cell surface markers CD31 (PECAM-1) and CD144 (VE-cadherin) of hUCB-EPCs were analyzed by flow cytometry (Fig. 7B). Interestingly, these results showed that Ac4ManNAz does not affect the natural characteristics of hUCB-EPCs.

## Discussion

Stem cell-based therapy holds great promise for repairing damage and injury of the human body; however, the safety and efficacy of stem cell *in vivo* conditions have not been fully confirmed^50^. Additionally, although hUCB-EPCs have great potential as therapeutics for cardiovascular diseases including coronary artery disease and stroke, the roles, behavior and fate of hUCB-EPCs *in vivo* are not completely understood^51^. Many cell labeling and tracking methods have been used and developed for hUCB-EPCs safety and efficacy *in vivo*^17^. Metabolic labeling is one of the most powerful methods for cell labeling and tracking and has proven to be superior to other imaging approaches^29,30^. However, metabolic labeling has not yet been widely used in stem cell tracking, because the introduction of abiotic azido groups in native proteins via PTM can potentially affect cell signaling pathways, cell adhesion, cell proliferation and the immune response. We previously reported the effects of metabolic labeling agents on cancer cells, showing that a higher concentration of Ac4ManNAz, which was recommended to achieve the best labeling efficiency, led to inhibition of basic cellular properties and down-regulation of many genes related to cell biological functions^52^. Therefore, to successfully apply the metabolic labeling technique to EPC tracking and labeling, the effects of metabolic labeling agents must be demonstrated. Thus, in this study, we reported for the first time the effects and optimized conditions of a metabolic labeling agent in hUCB-EPCs.

Metabolic labeling agents are known to have three type of azido sugars (Ac4ManNAz, Ac4GalNAz and Ac4GlcNAz), which introduce the azido group onto a native protein using different glycosylation processes. Thus, we tested all azido sugars to screen for optimal metabolic labeling agents for hUCB-EPCs. As a result, treatments with Ac4GalNAz and Ac4GlcNAz showed weaker metabolic labeling efficiency than Ac4ManNAz. In addition, Ac4GlcNAz had very low labeling efficiency. Generally, O-glycosylated proteins are highly abundant in mammalian cells^53^ and Ac4GalNAz and Ac4GlcNAz use the endogenous salvage pathway to introduce the azido group via O-linked glycosylation, which synthesizes UDP-GalNAc and GlcNAc from GalNAc and GlcNAc. Although these modification pathways involving numerous proteins, including various enzymes and nuclear pore proteins, are well-defined, conjugating an azido group on GalNAc and GlcNAc could be affected by enzyme function^54^. Indeed, the Bertozzi research group has shown that AGX1 encodes UDP-GlcNAc pyrophosphorylase, which is the final enzyme in UDP-GalNAc and GlcNAc biosynthesis and has the greatest loss of catalytic efficiency with azide-functionalized substrate analogs^55,56^. However, our study was focused on the optimization of hUCB-EPC labeling using the metabolic labeling technique. Thus, we finally selected Ac4ManNAz.

In the manufacturer’s procedure, the treatment of 50 μM Ac4ManNAz as maximum level was recommended for the highest labeling efficiency. Also, many studies of cell or virus labeling and tracking was normally used in Ac4ManNAz concentration of 50 μM or more^57,58^. However, in our results, the decreased proliferation rate, viability, rate of endocytosis and induction of ROS generation and mitochondrial depolarization were caused by high concentration of Ac4ManNAz. The metabolic labeling agent Ac4ManNAz is composed of a sugar molecule (ManNAc) and an azido group, which are incorporated into cellular proteins. Therefore, Ac4ManNAz has the potential to affect metabolic flux and cellular mechanisms by addition of sugar molecules. Regarding this, in previous reports, a high concentration (1 mM) of ManNAc treatment in mouse ESCs induced switching of epigenetic factors from Sirt1/Ogt to Mgea5 at the Hcrt gene locus^59^. A high concentration (>1,000 µM) of Ac4ManNAc, which is a ManNAc analog without the azido group, dramatically increased cytotoxicity by inducing the accumulation of acetic acid in cells^60^. Additionally, we previously analyzed the effects of ManNAc treatment on the A549 cancer cell line, ManNAc treatment at the same concentration of metabolic labeling agents does not affect cellular function or proliferation^52^. Also, the carbon energy source in the EGM-2-MV BulletKit Medium is the 5 mM. Ac4ManNAz were used less than 50 μM, which is a lower concentration by 100-fold. So, the addition of Ac4ManNAz as carbon energy source shown the negligible effects. These reports suggested that, changes of cellular physiology and mechanisms on hUCB-EPCs were caused by introduction of the azido group derived from Ac4ManNAz onto a native protein.

Non-toxic labeling method of azide group was the strain-promoted azide alkyne click chemistry (SPAAC) reaction, which rely on the use of cyclooctynes derivatives such as bicyclo[6.1.0]nonyne (BCN), difluorooctyne (DIFO), dibenzylcyclooctyne (DIBO) and DBCO^61^. These cyclooctynes derivatives have high selectivity of azide-functionalized biomolecules. However, several groups have recently reported that thiol-reactive group (-SH) can be conjugated with various cyclooctyne derivatives in the presence of high thiol concentrations as thiol-yne reaction, which can lead to issues associated with nonspecific background labeling and affected the efficiency of biomolecules^62–64^. Nevertheless, in mammalian proteins, the occurrence frequency of cysteine targets, which present the thiol group, is 3.3% and SPAAC reaction have the significantly higher reaction rates compared to thiol-yne reactions. For example, the BCN–azide reaction (10^−1^ m^−1^ s^−1^) is approximately three orders of magnitude greater than the rate constant for the BCN–thiol reaction (10^−4^ m^−1^ s^−1^)^64–66^. In addition, Chin Fen Teo *et al*. optimized the ratio of the reaction between azido-DBCO functionalities to the formation of thiol-DBCO product. These results shown that the reaction to proceed longer than 1 hour leads to increase the non-specific thiol-DBCO reaction and azido-DBCO specific reaction reaches completion within 1 hour^67^. Based on these results, in our experiments, hUCB-EPCs were treated with metabolic labeling agents supplemented medium (10, 20 and 50 µM) for 72 hours and all metabolic labeling agents-treated hUCB-EPCs were incubated with DBCO-Cy5 (10 µM, final concentration) for 1 hour at 37 °C. Thus, our optimized labeling procedures can be provided when highly specific labeling of hUCB-EPCs is required, thereby reducing the artifacts.

Our results reveal that the highly modified glycosylation by Ac4ManNAz treatments in hUCB-EPCs lead to a change in the physiological and biochemical properties of these cells. Notably, treatments with >20 µM Ac4ManNAz resulted in decreased growth rate, viability and rate of endocytosis and induction of ROS generation and mitochondrial depolarization. These results were consistent with a change in the expression of genes related to cell adhesion. Higher concentrations of Ac4ManNAz down-regulated the expression of integrin, collagen and fibronectin. Generally, integrins play an important role in various cell-signaling events including PI3K/AKT and FAK signaling pathways^68^, which help to regulate stem cell proliferation and self-renewal^69^. Accordingly, genes related to the PI3K/AKT, FGF and EGFR signaling pathways, which regulated cell proliferation, apoptosis, survival and immune response, were down-regulated^46,70^. Interestingly, expression of cadherins, which are cell adhesion molecules that actively contribute to cell death, survival and proliferation, was not inhibited by Ac4ManNAz treatments. The characterization of hUCB-EPCs were also not affected. Although treatments with >20 µM Ac4ManNAz led to negative outcomes in hUCB-EPCs, the effects of treatments with control or 10 µM Ac4ManNAz were not remarkably different. Additionally, it appears that 10 µM Ac4ManNAz provides sufficient labeling for tracking and monitoring hUCB-EPCs (Fig. S2). Thus, we emphasize the treatment of 10 µM Ac4ManNAz for labeling and tracking hUCB-EPCs as the optimal concentration because this concentration optimizes the proliferative capacity and functional property that are important for maximizing the use of EPCs as therapeutic agents.

In conclusion, this study described the optimal condition for the metabolic labeling technique for efficient labeling and tracking of hUCB-EPCs and the effects of metabolic labeling agents. Although high concentrations of Ac4ManNAz negatively affected hUCB-EPC properties, cell adhesion and cellular signaling pathways, 10 μM Ac4ManNAz showed the least adverse effects when compared to untreated hUCB-EPCs and this concentration provided sufficient labeling efficiency for cell labeling and tracking. Based on our results, we suggest 10 μM as the optimal concentration of Ac4ManNAz for *in vivo* cell labeling and tracking of hUCB-EPCs. Additionally, we expect that our approach can be used for understanding the efficacy and safety of stem cell-based therapy *in vivo* and to help determine the utility of stem cells in downstream experiments.

## Methods

### Ethic statement

All experiments were conducted in compliance with the relevant laws and institutional guidelines of the Korea Institute of Toxicology. The protocol was approved by the Committee on Biological Research of Korea Institute of Toxicology and Institutional Review Board (P01-201509-41-001).

### hUCB-EPCs culture

hUCB-EPCs were purchased from AllCells (Alameda, CA, USA). hUCB-EPCs culture were performed as previously described^71,72^. Briefly, mononuclear cells (MNCs) were first isolated from fresh hUCB-EPCs by density gradient centrifugation using Ficoll reagent (GE Healthcare, Piscataway, NJ, USA). MNCs were plated on fibronectin-coated tissue culture plates at a density of 3 ~ 6 × 10^6^ cells/6 wells in EGM-2-MV BulletKit Medium (Lonza, Walkersville, MD, USA) and cells were maintained for 5 ~ 7 days and used as an enriched EPC population.

### *In vitro* cell labeling and imaging

hUCB-EPCs (5 × 10^4^ cells/35 mm glass-bottom dishes) were treated with Ac4ManNAz, Ac4GalNAz, or Ac4GlcNAz (Invitrogen, Carlsbad, CA, USA) supplemented medium (50 µM, final concentration of each) for 72 h. Cells were washed twice with Dulbecco’s phosphate-buffered saline (DPBS) and subsequently incubated with DBCO-Cy5 (10 µM, final concentration) for 1 h at 37 °C. Cells were then washed and fixed with 4% paraformaldehyde for 15 min. After fixation, nuclei were stained with DAPI solution (Invitrogen, Carlsbad, CA, USA). All cell images were obtained using a confocal laser scanning microscope (Leica Microsystems, Mannheim, Germany) equipped with a 405 diode (405 nm) and HeNe-Red (633 nm) lasers.

### Western blot analysis

To confirm the introduction of azide (−N^3^) in the hUCB-EPCs, each 10–50 µM of Ac4ManNAz-, Ac4GalNAz-, or Ac4GlcNAz-treated hUCB-EPCs (2 × 10^6^ cells/100 mm dish) were prepared. Cells were washed twice with DPBS, pH 7.4 and harvested to extract cellular protein. A subcellular protein fractionation kit (Thermo Fisher Scientific, Rockford, IL, USA) was used according to the manufacturer’s instructions for segregating proteins from different cellular compartments including cytosol, membrane, nucleus, chromatin and cytoskeletons. Protein lysate concentrations were individually determined by bicinchoninic acid (BCA) protein assay (Thermo Fisher Scientific, Rockford, IL, USA) and protein concentrations were adjusted to 1 mg/mL. Then, 100 µL of lysate was incubated with 5 mM phosphine-PEG3-biotin in DPBS for 6 h at 37 °C to assess specific interactions between phosphine and an azide group of the cellular protein. Samples were boiled with SDS loading buffer and resolved by 10% SPS-PAGE and proteins were transferred to Hybond-P membrane (Amersham, St. Albans, UK). After blocking with 5% bovine serum albumin in TBST (50 mM Tris∙HCl, 150 mM NaCl, 0.1% Tween-20, pH 7.4), the membrane was incubated in streptavidin-HRP (diluted 1:10,000 in TBST) overnight at 4 °C. Then, the membrane was washed three times with TBST and developed using ECL Western Blotting Substrate (Thermo Fisher Scientific, Rockford, IL, USA).

### Cell viability and wound healing assay

To measure cell viability, hUCB-EPCs were seeded in 96-well plates (5 × 10^3^ cells/well) and incubated for 1 day. Cells were incubated with various concentrations of Ac4MAnNAz (0 to 50 µM) for 3 days at 37 °C. Cell Counting Kit-8 solution (10 µL) (Dojindo Molecular Technologies Inc., Kumamoto, Japan) was then added to each well. After further incubation for 2 h at 37 °C, the absorbance of each well was measured at 450 nm using a microplate reader (VersaMax^TM^, Molecular Devices Corp., Sunnyvale, CA, USA). For wound healing, a sterile pipette tip was used to clear a small area across the diameter of 10 cm dishes with confluent monolayers of untreated or Ac4MAnNAz-treated hUCB-EPCs. Cell migration was measured and photographed from the wound/scratch edge after 18 h.

### Analysis of reactive oxygen species (ROS) generation and mitochondrial membrane potential

Microscopic fluorescence imaging was used to study reactive oxygen species (ROS) generation in hUCB-EPCs after treatments to different concentrations of Ac4ManNAz. Cells (1 × 10^4^ per well) were seeded and were then treated to 0 µM, 10 µM, 20 µM and 50 µM concentrations of Ac4ManNAz for 3 days at 37 °C. Cells were incubated with 2,7-Dichlorodihydrofluorescein diacetate (DCF-DA) (10 mM) for 30 min at 37 °C. The reaction mixture was aspirated and replaced by 200 µl of phosphate-buffered saline (PBS) in each well. The plate was kept on a shaker for 10 min at room temperature in the dark. An inverted fluorescent microscope was used to visualize intracellular fluorescence of cells and to capture images. Mitochondrial membrane potential was measured using JC-1 dye (5′,6,6′-tetrachloro-1,1′,3,3′-tetraethylbenzimidazolyl-carbocyanine iodide; Life Technologies, Eugen, Oregon, USA) according to the manufacturer’s instructions. Briefly, hUCB-EPCs were grown in 24-well plate and treated with different concentrations of Ac4ManNAz. Ac4MAnNAz-treated or untreated hUCB-EPCs were washed with PBS and stained with 10 µg/mL JC-1 dye for 15 min at 37 °C and fluorescence images were acquired.

### Transfection and Qdot 525 labeling

hUCB-EPCs were transfected with 100 ng of pcDNA3-EGFP using Lipofectamine 2000 (Invitrogen, Carlsbad, CA, USA) according to the manufacturer’s instructions. In addition, 1 μM Qdot 525 (Thermo Fisher Scientific, Rockford, IL, USA) in medium was incubated for 4–6 h at 37 °C and hUCB-EPC samples were diluted in DPBS immediately before measurement. Then, we analyzed samples using fluorescence microscopy.

### RNA-seq analysis

hUCB-EPCs were cultured with 0, 10 or 50 μM Ac4ManNAz and harvested. Total RNA was extracted with TRIzol reagent (Invitrogen, Carlsbad, CA, USA) and the quantity and quality of total RNA were evaluated using a NanoDrop spectrophotometer (NanoDrop Technologies, Montchanin, DE, USA) and a 2100 Bioanalyzer (Agilent Technologies, Palo Alto, CA, USA). An RNA sequencing library was generated using TruSeq RNA Library Preparation Kit (Illumina, San Diego, CA, USA) according to the user’s instruction manual. Briefly, mRNA was separated from total RNA using Oligo (dT) beads and chemically fragmented. After double-strand cDNA synthesis of the fragmented mRNA, end-repair, adenylation of the 3′-end and sequencing adapter ligation were performed and followed by DNA purification with magnetic beads and PCR amplification. Finally, the amplified library was purified, quantified and then applied for template preparation. A HiSeq2000 platform was used to generate 99-bp paired-end sequencing reads (Illumina, San Diego, CA, USA). All 99-bp paired-end sequence reads were mapped to the human genome using TopHat version 2.0.4. Finally, we identified differentially expressed genes (DEgenes). To characterize the biological pathways related to differentially expressed sequences and transcription factors, representative pathways were analyzed in the context of several databases such as KEGG (http://www.genome.ad.jp), BioCarta (http://www.biocarta.com) and Reactome (http://www.reactome.org), as suggested by MsigDB v4.0. Additionally, we used Fisher’s exact test and FDR to examine mapping pathways (filtering options: *p* < 0.05 and FDR < 5). To identify cellular functional process-related genes, the DEgenes were subjected to gene ontology (GO) analysis using the Database for Annotation, Visualization and Integrated Discovery (DAVID), KEGG, BIOCARTA, REACTOME and Pathway Interaction Database (PID). We used a Gene Set Enrichment Analysis technique to identify statistically up- and down-regulated gene set.

### *In vitro* tube formation assay

Functional characterization of hUCB-EPCs was performed using vascular tube formation and acetylated low-density lipoprotein (ac-LDL; Invitrogen, Carlsbad, CA, USA) uptake assays. For the vascular tube formation assay, 50 μL of Matrigel Basement Membrane Matrix (BD Biosciences, San Diego, CA, USA) was added to a 48-well plate and allowed to solidify at 37 °C for 30 min; 5 × 10^4^ hUCB-EPCs were suspended in 100 μL of culture medium and plated onto the Matrigel layer. After 24 h, the medium was removed and the formation of vascular tube-like structures was assessed with an inverted microscope (Eclipse TS100; Nikon, Tokyo, Japan) and a digital camera system for imaging (Digital SLR Camera D300; Nikon, Tokyo, Japan). For the ac-LDL uptake assay, hUCB-EPCs were seeded onto glass coverslips in 24-well plates at a density of 5 × 10^4^ cells/well in MV2 medium. When cells reached ∼80% confluence, cultures were serum-deprived overnight in Iscove’s Modified Dulbecco’s Medium (IMDM, Sigma-Aldrich, St. Louis, MO, USA) supplemented with 2% lipoprotein-deficient serum from human plasma (Sigma-Aldrich, St. Louis, MO, USA) and then, the medium was replaced with IMDM supplemented with 100 μg/mL human ac-LDL. After 24 h, cells on coverslips were stained with Nile Red (Sigma-Aldrich, St. Louis, MO, USA) and examined by fluorescence microscopy with an inverted microscope (Eclipse TS100; Nikon, Tokyo, Japan) and a digital camera system for imaging (Digital SLR Camera D300; Nikon, Tokyo, Japan).

### Flow Cytometry

To analyze hUCB-EPC marker genes, immunophenotyping was performed using the following monoclonal antibodies: anti-CD31-FITC (1:25, BD Biosciences, San Jose, CA, USA) and anti-CD144-PE (1:10, Beckman Coulter, Fullerton, CA, USA). Antibodies and matched isotype control (Beckman Coulter, Fullerton, CA, USA) were incubated for 30 min at 4 °C. Data were acquired and analyzed on a five-parameter flow cytometer (FACSCalibur, Becton Dickinson, San Jose, CA) with Weasel software (WEHI, Melbourne, Australia).

### Statistical analysis

Experimental data are presented as a mean ± standard deviation and were analyzed using Analysis of Variance (ANOVA) tests. A value of *p* < 0.05 was considered statistically significant.

## Electronic supplementary material

Supporting Information
