# Supplementary material for: Safety and Optimization of Metabolic Labeling of Endothelial Progenitor Cells for Tracking
Source: Sci Rep. 2018 Sep 4;8:13212. doi: 10.1038/s41598-018-31594-0 (PMC6123424; doi:10.1038/s41598-018-31594-0)

Supporting Information

**Safety and Optimization of Metabolic Labeling of Endothelial Progenitor Cells for Tracking**

Sang-Soo Han^1*^, Hye-Eun Shim^1*^, Soon-Jung Park^2^, Byoung-Chul Kim^3^, Dong-Eun Lee^4^, Hyung-Min Chung^2^, Sung-Hwan Moon^2#^, Sun-Woong Kang^1,5#^

^1^Predictive Model Research Center, Korea Institute of Toxicology, Daejeon, Korea, ^2^Department of Stem Cell Biology, School of Medicine, Konkuk University, Seoul, Korea, ^3^The Genomics Institute, Ulsan National Institute of Science and Technology, Ulsan, Korea, ^4^Advanced Radiation Technology Institute, Korea Atomic Energy Research Institute, Jeonbuk, Korea, ^5^Department of Human and Environmental Toxicology, University of Science and Technology, Daejeon, Korea

**Figure S1**. **Cell apoptosis assays with an Annexin V-FITC Apoptosis Detection Kit.** The effects of higher levels of Ac4ManNAz, Ac4GalNAz, or Ac4GlcNAz by Annexin V staining were evaluated using a FITC Annexin V apoptosis detection kit. hUCB-EPCs (5 × 10^4^ cells/35 mm glass-bottom dishes) were treated with Ac4ManNAz, Ac4GalNAz, or Ac4GlcNAz supplemented medium (0, 10, 20, and 50 µM, final concentration of each) for 72 h. After incubation, cells were analyzed by flow cytometry using FACS and the Cellquest Pro software.


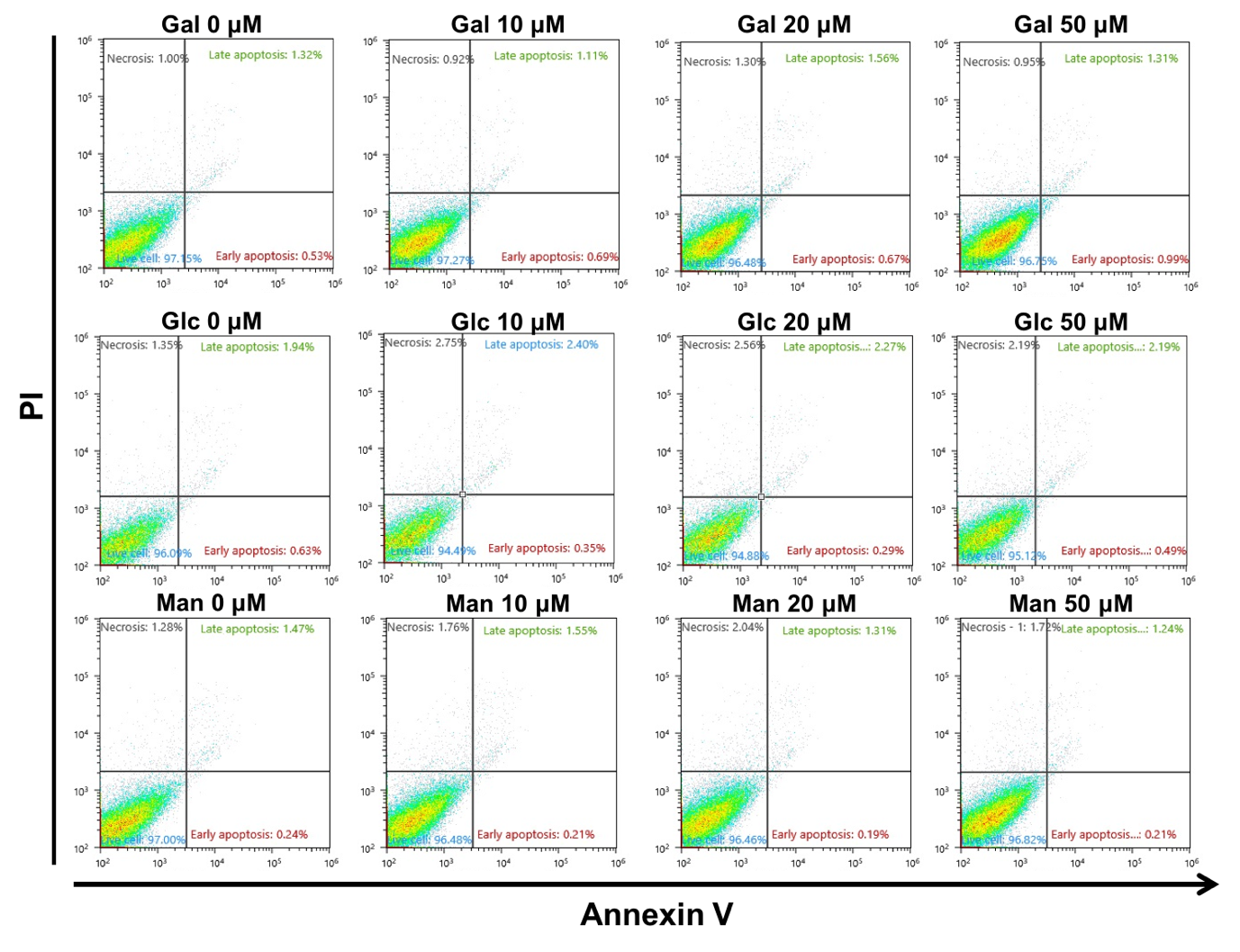


**Figure S2**. **Comparative analysis of Ac4ManNAz concentration-dependent hUCB-EPC labeling efficiency.** (A) Confocal microscopic images of Ac4ManNAz (0-50 µM)-labeled hUCB-EPCs. (B) FACs analysis for fluorescence intensities of Ac4ManNAz (0-50 µM)-labeled hUCB-EPCs


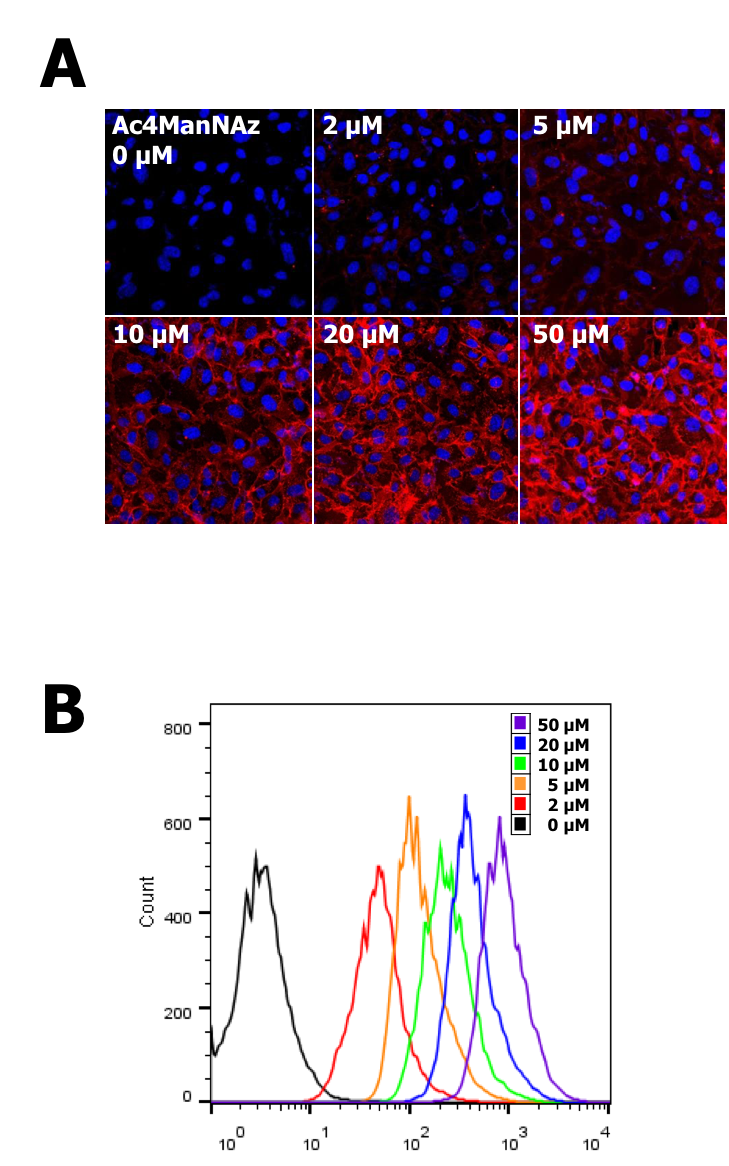

Supplement: Supplementary file 1 — Supporting Information [file 41598_2018_31594_MOESM1_ESM.docx]
